# Theoretical, Technical, and Analytical Foundations of Task-Based and Resting-State Functional Magnetic Resonance Imaging (fMRI)—A Narrative Review

**DOI:** 10.3390/biomedicines14020333

**Published:** 2026-01-31

**Authors:** Natalia Anna Koc, Maurycy Rakowski, Anna Dębska, Bartosz Szmyd, Agata Zawadzka, Karol Zaczkowski, Małgorzata Podstawka, Dagmara Wilmańska, Adam Dobek, Ludomir Stefańczyk, Dariusz Jan Jaskólski, Karol Wiśniewski

**Affiliations:** 1 Faculty of Medicine, Medical University of Gdańsk, 80-210 Gdańsk, Poland; 2 Department of Neurosurgery and Neurooncology, Barlicki University Hospital, Medical University of Łódź, 90-153 Łódź, Polanddariusz.jaskolski@umed.lodz.pl (D.J.J.);; 3 Siemens Healthcare Sp. z o.o., 03-821 Warsaw, Poland; 4 Department of Radiology and Diagnostic Imaging, Barlicki University Hospital, Medical University of Łódź, 90-153 Łódź, Polandludomir.stefanczyk@umed.lodz.pl (L.S.)

**Keywords:** resting-state fMRI, task-based fMRI, neurosurgical planning, brain mapping, functional connectivity, presurgical mapping

## Abstract

Functional magnetic resonance imaging (fMRI) is a valuable tool for presurgical brain mapping, traditionally implemented with task-based paradigms (tb-fMRI) that measure blood oxygenation level-dependent (BOLD) signal changes during controlled motor or cognitive tasks. Tb-fMRI is a well-established tool for non-invasive localization of cortical eloquent areas, yet its dependence on patient cooperation and intact cognition limits use in individuals with aphasia, cognitive impairment, or in pediatric and other vulnerable populations. Resting-state fMRI (rs-fMRI) provides a task-free alternative by leveraging spontaneous low-frequency BOLD fluctuations to delineate intrinsic functional networks, including motor and language systems that show good spatial concordance with tb-fMRI and with direct cortical stimulation. This narrative review outlines the methodological foundations of tb-fMRI and rs-fMRI, comparing acquisition protocols, preprocessing and denoising pipelines, analytic approaches, and validation strategies relevant to presurgical planning. Particular emphasis is given to the technical and physiological foundations of BOLD imaging, statistical modeling, and the influence of motion, noise, and standardization on data reliability. Emerging evidence indicates that rs-fMRI can reliably expand mapping to patients with limited task compliance and may serve as a robust complementary modality in complex clinical contexts, though its methodological heterogeneity and absence of unified practice guidelines currently constrain widespread adoption. Future advances in harmonized preprocessing, multicenter validation, and integration with connectomics and machine learning frameworks are likely to be critical for translating rs-fMRI into routine, reliable presurgical workflows.

## 1. Introduction

Functional magnetic resonance imaging (fMRI) is a non-invasive neuroimaging method that measures brain activity by detecting changes in blood oxygenation and flow associated with neural activation [1]. Unlike structural MRI, which provides anatomical information, fMRI captures dynamic physiological changes, allowing for the mapping of areas involved in sensory, cognitive, and motor functions [2]. The technique is based on the blood oxygenation level-dependent (BOLD) signal, which reflects changes in cerebral blood flow (CBF), volume, and oxygen metabolism associated with neural activity [3]. Localized neuronal activation increases metabolic demand and triggers a supply of oxygenated blood, reducing the relative concentration of paramagnetic deoxygenated hemoglobin (dHb) and producing a detectable T_2_*-weighted MR signal. The hemodynamic response follows a characteristic temporal pattern, peaking approximately 4–6 s after neural activation and returning to baseline after about 12–20 s [4]. Although fMRI does not measure neuronal firing directly, the resulting hemodynamic response provides a reliable proxy for brain activity, offering reasonable spatial and acceptable temporal resolution and making fMRI an essential tool in both cognitive neuroscience and clinical brain mapping [5].

There are two main modes of fMRI acquisition: task-based (tb-fMRI) and resting-state (rs-fMRI). Tb-fMRI relies on controlled paradigms where subjects perform specific cognitive or motor tasks to evoke localized brain activation, which is then statistically modeled using the general linear model (GLM) [6]. Rs-fMRI measures spontaneous low-frequency (<0.1 Hz) fluctuations in the BOLD signal while the subject is not performing any explicit task, allowing analysis of intrinsic functional connectivity between brain regions [7]. Both modalities depend on the same physiological principle but differ in their methodological approach and clinical applicability. Tb-fMRI, often used for precise localization of eloquent cortical areas, such as motor, sensory, or language regions, is relatively resilient to random motion artifacts because the task provides a temporal anchor against which unrelated fluctuations can be filtered [8]. However, its efficacy depends on the patient’s ability to understand, cooperate, and execute the required tasks, which can limit its use in populations with neurological deficits, cognitive impairments, pediatric patients, and those unable to comply with instructions. This may yield false-negative or unreliable activation maps, potentially leading to mislocalization of critical areas, which increases the risk of postoperative deficits or constrain surgical options due to uncertain functional boundaries [6]. Rs-fMRI, by contrast, provides a task-independent approach that leverages intrinsic brain connectivity, offering critical complementary information when task-based methods are infeasible or unreliable [7]. It boasts a lower failure rate since it does not rely on task performance, but is highly sensitive to subtle motion artifacts, which can be misinterpreted as functional connectivity. In this sense, rs-fMRI requires extensive post-processing to remove confounding factors such as respiration, cardiac pulsations, or head motion that could masquerade as genuine connectivity [9].

Overall, while tb-fMRI remains the clinical standard for non-invasive functional brain mapping, rs-fMRI is emerging as a powerful complementary technique—and in some cases, a viable substitute—particularly when traditional paradigm-driven approaches are not feasible. Understanding the trade-offs between resilience to motion, dependency on task performance, and signal interpretability is essential for optimizing their use in different clinical scenarios. This narrative review aims to provide comprehensive exploration of the theoretical and methodological foundations of both fMRI modalities, with a focus on the translational implications of rs-fMRI as an adjunct or alternative to tb-fMRI in preoperative brain mapping. By offering a structured comparison of acquisition methods, signal characteristics, patient considerations, and analytical strategies, we aim to provide a solid basis for understanding how these tools can be deployed in research and clinical settings. Furthermore, we highlight emerging trends and practical challenges involved in integrating rs-fMRI into standard neurosurgical workflows, offering clinicians and researchers a framework for informed application of these techniques in contemporary neuroimaging practice.

## 2. The BOLD Mechanism

### 2.1. Hemodynamic Response and Neural Activity Coupling

Both structural MRI and fMRI share the same physical foundation: the detection of signals generated by hydrogen nuclei (protons) abundant in biological tissue. When placed in a strong static magnetic field (B_0_), these spins align along the field and precess at a frequency proportional to the field strength. A brief radiofrequency pulse tips this magnetization away from equilibrium, inducing a measurable transverse signal that gradually decays as the spins lose phase coherence. Decay occurs through two relaxation processes: T_1_ (longitudinal recovery) and T_2_ (transverse decay). While structural MRI primarily exploits T_1_ differences among tissues, fMRI is based on the T_2_*-weighted contrast, which is sensitive to changes in local magnetic susceptibility caused by variations in hemoglobin oxygenation [4].

The BOLD effect was first demonstrated by Ogawa and colleagues in 1990, who observed that the MR signal varied with blood oxygenation in animal brain tissue. They attributed these changes to susceptibility effects caused by dHb within red blood cells [10]. Local variations arise because dHb is paramagnetic, whereas oxygenated hemoglobin (Hb) is diamagnetic. The increase in dHb concentration distorts the surrounding magnetic field, accelerating T_2_* decay and thereby reducing the measured MR signal intensity, while the decrease in dHb causes an increase in image intensity [5]. Neural activation initiates a cascade of tightly coordinated physiological responses involving changes in CBF, cerebral blood volume (CBV), and the cerebral metabolic rate of oxygen consumption (CMRO_2_), which forms the basis of the classic triphasic BOLD response: the initial dip, the main positive peak, and the post-stimulus undershoot [4,5]. During localized neuronal activation, energy demand increases, initially raising oxygen consumption and transiently elevating dHb concentration, producing a brief “initial dip” in signal intensity. Within approximately two seconds, a large overcompensatory rise in CBF delivers more oxygenated blood than is consumed, flushing out dHb and causing a slower T_2_* decay, which is detected as a small local increase in the signal, known as BOLD signal [4]. Quantitatively, CBF increases by 30–50%, while CMRO_2_ increases by 10–15%, resulting in a net decrease in the oxygen extraction fraction (OEF)—a fraction of O_2_ carried by an element of blood that is removed in passing through the capillary bed. CBV increases concurrently by approximately 10%, though its temporal evolution is slower than that of CBF, causing decoupling critical for the post-stimulus undershoot phase. These physiological shifts typically yield a BOLD signal change on the order of 0.6–0.8% of baseline intensity, necessitating the optimization of image acquisition to distinguish neural signals from physiological noise [3]. The HRF describes the temporal profile of this vascular response, with the main peak occurring 4–6 s after neuronal activation and post-stimulus undershoot lasting even over 30 s. However, interindividual, interregional, and stimulus-dependent factors can substantially alter the timing, morphology, and amplitude of the BOLD response [11]. Advancements in HRF modeling suggest moving beyond fixed scalar values toward data-driven, smooth modeling approaches, which allow voxel-wise HRF estimation constrained by physiological plausibility (e.g., using thin plate splines and roughness penalties). This improves sensitivity and permits more accurate inferences about neuronal activity, especially in clinical populations or group comparisons where standard models may mischaracterize timing by up to 1–2 s [12]. In rs-fMRI, the same mechanism underlies the detection of spontaneous, low-frequency fluctuations (<0.1 Hz) in the BOLD signal, which are temporally correlated between functionally connected regions, forming the basis of intrinsic connectivity networks [7]. Regions that exhibit synchronized BOLD fluctuations are considered to be functionally connected and form resting-state networks (RSNs), such as the Default Mode Network (DMN) or Sensorimotor Network (SMN). The measurement of these temporally correlated fluctuations forms the entire basis for investigating the brain’s intrinsic functional architecture [13].

The BOLD signal integrates the changes in flow, volume, and metabolism, representing a composite vascular response to neural metabolic demand rather than directly measuring neural firing. From a neurophysiological standpoint, comparisons between fMRI and electrophysiology indicate that the BOLD signal correlates more strongly with local field potentials (LFPs), which suggests that fMRI primarily maps brain regions based on the energy required for synaptic communication and sub-threshold processing. The BOLD response is less correlated with Multiunit Activity (MUA), which represents the frequency of neuronal action potentials (spikes) used for long-distance communication [14]. Experiments demonstrating that suppression of MUA can occur without a corresponding change in the BOLD response suggest that the energy consumption associated with spiking activity is a small component of the total metabolic demand measured by fMRI [14,15]. Therefore, the BOLD signal effectively highlights the metabolic cost of incoming information and local integration. The biological mechanisms underlying this coupling seem to involve interactions between neurons, astrocytes, and vascular smooth muscle cells. Upon synaptic activation, glutamate, the primary excitatory neurotransmitter, is released and subsequently taken up by neighboring astrocytes. This uptake is metabolically demanding, stimulating astrocytic glycolysis and triggering the release of vasoactive molecules such as nitric oxide (NO), adenosine, and prostaglandins, which act on local arterioles, inducing vasodilation and increasing regional CBF during the overshoot phase of the BOLD response [5,7]. In the undershoot phase, the balloon model characterizes venous compartments as distensible vessels, causing CBV to return to baseline more slowly than CBF. This temporal mismatch also seems to prolong the presence of dHb and contribute to the post-stimulus undershoot of the BOLD signal [11]. While traditionally interpreted as a mechanism for oxygen delivery, the increase in blood flow also seems to reflect glucose metabolism and neurotransmitter recycling, highlighting the metabolic complexity of the BOLD response.

### 2.2. Signal Susceptibility, Motion, and Noise

The BOLD contrast modulates the T_2_ signal primarily by reducing signal decay through activity-related decreases in paramagnetic dHb [4,10]. This effect scales approximately quadratically with the magnetic field strength, which reflects the dynamic averaging of diffusion in the presence of field gradients [4]. Because the functional BOLD signal change is typically only a few percent of the total image intensity, the central challenge in fMRI is achieving a sufficient signal-to-noise ratio (SNR) to reliably distinguish task-related brain activity from background physiological and scanner noise [5,14]. Modern fMRI studies address this by using high field strengths (3 T or above) and fast imaging sequences, such as gradient-echo echo-planar imaging (EPI), which are designed to maximize sensitivity to T_2_* changes and thus to enhance the functional signal. However, the very imaging sequences that maximize the BOLD signal’s sensitivity also introduce significant sources of noise and artifacts. The T_2_*-weighting that makes the sequence sensitive to dHb fluctuations also makes it highly sensitive to inhomogenities, which naturally occur at the interface between materials with different magnetic properties. The most severe artifacts are found near air-tissue boundaries, particularly the paranasal sinuses and ear canals [16]. Such boundaries introduce strong, localized magnetic field gradients that result in two major forms of data degradation: signal dropout and geometric distortion. In the presence of a strong inhomogeneity, the precession frequencies of water protons within a single voxel become spread out. This leads to rapid dephasing of the magnetic signal within the echo time, causing the T_2_* signal to effectively drop to zero. This occurs particularly in crucial regions for cognitive and affective processing, such as the orbitofrontal cortex and the inferior temporal lobes [4,16]. Strong field variations also cause spatial compression or stretching of the image, leading to a misregistration of the functional data onto the corresponding high-resolution anatomical scan, known as geometric distortion [16]. Distortion artifacts are further exacerbated by subject motion. Even micromovements change the head’s position relative to the main magnetic field, causing the pattern of field inhomogeneity to change dynamically. This complicates correction efforts and introduces spurious, high-amplitude signal changes that are entirely unrelated to the neural task, effectively reducing the SNR [4]. This necessitates sophisticated, multi-stage correction algorithms to align the data, though complete correction is rarely achieved [16]. Endogenous bodily functions, such as breathing or cardiac variability, also introduce systemic noise. Breathing causes not only bulk movements of the chest and abdomen but also modulates the concentration of CO_2_ in the blood [3]. Since CO_2_ is a powerful vasodilator, changes in respiratory depth or rate can cause global BOLD signal fluctuations by altering CBF across the entire brain, mimicking task-related activity, while the rhythmic flow of blood and cerebrospinal fluid (CSF) also creates periodic signal fluctuations that must be filtered out to improve the functional SNR [4].

## 3. Task-Based fMRI (Tb-fMRI)

### 3.1. Experimental Design: Block vs. Event-Related Paradigms

Tb-fMRI remains the most established non-invasive method for localizing functional brain areas in both research and clinical practice. It capitalizes on the BOLD signal to measure regional hemodynamic changes associated with neural activation during task performance. The resulting activation maps have become indispensable in presurgical planning for patients with brain tumors, epilepsy, and vascular malformations, as well as in cognitive neuroscience for understanding the functional architecture of the human brain [1,2,17].

The design of the experimental paradigm critically determines the sensitivity, specificity, and interpretability of tb-fMRI results. Two main design frameworks are used: block designs and event-related designs, each offering distinct advantages depending on the research or clinical objective [6].

In a block design, the participant alternates between periods of task performance (activation blocks) and rest or control conditions (baseline blocks), typically lasting 20–40 s per phase. For example, a participant might be instructed to tap their fingers repeatedly for 30 s (task block), followed by 30 s of rest (control block). The BOLD signal in this paradigm is expected to rise sharply at the start of the task block, stay high (plateau) throughout the duration due to the temporal summation of responses, and fall back to baseline during the rest block, creating a predictable time series that can be modeled with high statistical power [18]. The temporal summation of hemodynamic responses across blocks enhances the SNR, making this design particularly well-suited for robust clinically oriented applications such as motor or language mapping in neurosurgical patients [19]. Its relative simplicity facilitates patient compliance and makes it the preferred approach in clinical fMRI centers. However, the prolonged nature of activation blocks can induce cognitive fatigue, potentially leading to a decline in participant motivation and performance as the scan progresses. These fluctuations in the participant’s internal state can introduce significant confounds, as the resulting fMRI signal may reflect a mixture of task performance and varying levels of engagement. Furthermore, block designs are generally poorly suited for capturing transient neural dynamics—such as those occurring specifically at the onset or offset of a task—because these brief signals are typically obscured by the broader plateau of the sustained BOLD response [20]. Event-related designs, in contrast, present individual stimuli or tasks as discrete events separated by a short interstimulus interval randomized in timing. The signal peaks around 6–9 s after each discrete event before returning to the baseline, allowing the estimation of the hemodynamic response to a single trial [1]. This design allows the estimation of the hemodynamic response to single events, providing greater flexibility to study transient or cognitive processes such as attention, error monitoring, and memory encoding. Event-related paradigms enable post hoc sorting of trials based on behavioral performance or stimulus characteristics, offering more precise modeling of cognitive operations. However, they require longer scan times and often yield lower statistical power, making them less practical in clinical populations [20]. An emerging approach is the mixed or hybrid blocked/event-related design. By embedding discrete events within longer blocks, these designs attempt to combine strengths, allowing researchers to model both sustained activity linked to the block (e.g., an attentional set) and transient activity linked to the individual event (e.g., a target stimulus) within a single experiment [20]. While complex, this approach offers a refined method for dissecting complex cognitive operations.

### 3.2. Role of Statistical Modeling

The goal of fMRI analysis is to determine which voxels exhibit a change in BOLD signal that is significantly related to the experimental task. Given that the recorded fMRI time series data is complex and contaminated by various noise sources, this inference requires a powerful statistical framework, the most common of which is the GLM, particularly for tb-fMRI studies [6,21]. The GLM is a voxel-wise regression technique applied independently to the BOLD time series of every voxel in the brain. Conceptually, the model posits that the observed BOLD signal, *Y*, is equal to a linear combination of explanatory variables, *X*, plus an additive error term, *ϵ*:

*Y* = *Xβ* + *ϵ*

In this equation:

*Y* represents the measured BOLD signal across all time points for a single voxel.
*X* is the design matrix, which includes the model of expected neural activity.
*β* is the vector of parameter weights that the model solves for. These values quantify the degree to which the observed BOLD signal is explained by each explanatory variable.

The most crucial step in constructing the design matrix (*X*) for tb-fMRI is accurately modeling the expected BOLD response. For standard analyses, the task design matrix, encoding stimulus timing and duration, is convolved with a canonical HRF to approximate the expected BOLD response. This matrix also includes covariates of no interest, such as motion parameters, to model and remove noise [21]. After the GLM is fitted to the data, the parameter estimates (*β*) are used to test specific hypotheses via statistical contrasts, typically *t*-tests or F-tests, to determine if the effect is significantly greater than the null hypothesis of no activation [22]. To characterize the finer temporal structure of the response, especially when the image acquisition time is comparable to the hemodynamic latency, advanced models may employ temporal basis functions, such as Fourier series. This technique allows for improved temporal resolution by varying the phase of the stimulus relative to the acquisition, while the GLM provides unbiased and least squares estimates for the response’s coefficients [21]. The process of generating comparisons for every voxel in the brain results in a continuous image known as a statistical parametric mapping (SPM) that displays regions of significant activation, typically overlaid on high-resolution anatomical images [23]. With tens of thousands of voxel-wise tests, the overall familywise error rate (FWER) is severely inflated, leading to many false positives. To control for this, the raw SPMs must undergo rigorous correction methods. Standard approaches include family-wise error (FWE) that corrects the significance threshold so that the probability of one or more false positives across the entire search volume remains below a set ɑ value. Less conservative approaches include a false discovery rate (FDR) error metric, which controls the expected proportion of false positives among all declared active voxels, and cluster-based thresholding, which uses the spatial smoothness of the fMRI data, where voxels must belong to a contiguous cluster of a minimum predefined size to be considered significant [22]. While the GLM remains the standard for the tb-fMRI modeling, multivariate approaches like partial least squares (PLS) can also be employed to assess task-related connectivity between distributed regions, offering an alternative perspective on network engagement during active tasks [24]. In presurgical contexts, these activation maps are often exported into neuronavigation systems to guide surgical planning and minimize postoperative deficits. Validation studies have shown good concordance between tb-fMRI activations and direct cortical stimulation (DCS) results, particularly for motor mapping, where fMRI has shown sensitivity of up to 85% [25,26]. However, for language areas, tb-fMRI tends to produce more diffuse activation patterns than rs-fMRI, reflecting responses beyond language-specific cortex due to the involvement of domain-general networks [26]. For language mapping, sensitivity varies more widely, with estimates ranging from 59% to 100%, and specificity from 0% to 97% across studies, prompting caution in relying solely on fMRI to identify language-eloquent regions [25].

### 3.3. Example Paradigms

The choice of tb-fMRI paradigm is driven by the functional system of interest and the clinical or research objectives. Carefully selected paradigms reliably activate target brain regions, facilitating accurate functional localization. Motor paradigms (e.g., finger tapping, hand grasping, foot dorsiflexion) reliably activate the primary motor cortex, supplementary motor area, and contralateral cerebellum, producing strong, reproducible BOLD responses make them well-suited for presurgical motor mapping [27]. Language paradigms such as verb generation, picture naming, and semantic decision tasks are designed to activate classical language processing areas, including Broca’s area in the inferior frontal gyrus and Wernicke’s area in the posterior temporal cortex [28]. These tasks provide critical information on hemispheric dominance for language and can guide surgical planning by delineating essential language areas. Comparative studies have shown that tb-fMRI results generally align well with DCS in terms of language lateralization and localization; however, task selection remains critical, as different paradigms can yield variable activation patterns [25,29]. Visual paradigms, including checkerboard pattern reversals or moving dot fields, elicit robust activation of the primary visual cortex and extrastriate regions, making them useful for baseline calibration and visual pathway assessment, which is especially important in evaluating lesions near the occipital lobe [30]. Other paradigms, such as auditory, working memory, and emotional processing tasks, have also been developed, but their use in routine clinical practice remains limited due to longer acquisition times and the need for active cognitive engagement.

### 3.4. Dependence on Subject Compliance and Task Performance

A major limitation of tb-fMRI is its reliance on patient cooperation and consistent task execution. This technique fundamentally assumes that participants perform the tasks as instructed, which may not be feasible for many neurological patients, especially those with aphasia, motor deficits, cognitive impairment, or altered consciousness [30]. Such patients often struggle with following instructions or maintaining task engagement, resulting in incomplete or misleading activation maps that could obscure critical eloquent brain regions. Variability in task performance introduces substantial within-subject and between-subject noise, degrading the fit of the statistical model and reducing the sensitivity and specificity of detecting true brain activations [31]. Motion artifacts, common in patients with tumors, seizures, or pediatric populations, can further distort the BOLD signal and confound activation patterns [32,33]. While behavioral monitoring methods such as visual feedback, button presses, or real-time motion monitoring can mitigate some compliance issues, these tools are not uniformly available or practical in many clinical settings. Shortening scan times or simplifying paradigms might improve patient tolerance but can sacrifice data richness [31]. For populations unable to perform tasks, such as those with severe aphasia, altered consciousness, or pediatric patients, tb-fMRI becomes impractical. Rs-fMRI presents a task-free, alternative method to identify intrinsic functional networks without requiring active cooperation, offering an effective and often described as more sensitive and detailed solution in these challenging cases [26,32]. Consequently, many modern centers have adopted multimodal mapping protocols that integrate tb-fMRI and rs-fMRI to achieve comprehensive functional assessment and enhance surgical safety [34].

## 4. Resting-State fMRI (Rs-fMRI)

### 4.1. Concept of Spontaneous Low-Frequency Fluctuations

The concept of rs-fMRI emerged from the discovery that the brain exhibits coherent, spontaneous fluctuations in the BOLD signal even in the absence of an explicit task. In his seminal study, Biswal et al. demonstrated that slow fluctuations in the motor cortex were temporally correlated between functionally related regions, revealing intrinsic functional connectivity within neural networks [35]. Another evidence came from positron emission tomography (PET) studies, which showed that specific regions, particularly the posterior cingulate cortex (PCC) and ventral anterior cingulate cortex (vACC), consistently showed reduced activity during task performance across a wide range of cognitive domains [36,37]. This finding fundamentally shifted the use of fMRI from stimulus-driven activation mapping to the study of the brain’s intrinsic organization and baseline functional architecture [37].

Physiologically, these spontaneous low-frequency fluctuations (sLFFs) reflect synchronized modulations in neuronal and vascular activity across distributed brain regions. The low-frequency BOLD oscillations (typically between 0.01 and 0.1 Hz) differ from higher-frequency physiological signals such as cardiac pulsation (~1 Hz) or respiration (~0.3 Hz), corresponding to the intrinsic time constants of cerebrovascular regulation and neural network interactions [4]. They are believed to originate from ongoing neuronal oscillations and metabolic cycles that persist during wakeful rest, representing the brain’s default mode of operation rather than random noise [37,38]. Electrophysiology and optical imaging studies have shown that these slow BOLD oscillations correlate with fluctuations in local field potentials, particularly within infra-slow frequency bands linked to synaptic and glial activity [14,38]. Thus, rs-fMRI captures the aggregate hemodynamic consequences of ongoing, large-scale neural synchronization, sustained by continuous metabolic and vascular coupling even in the absence of explicit behavioral demands.

Methodologically, rs-fMRI isolates these intrinsic signals by recording continuous T_2_*-weighted BOLD data while subjects rest quietly, typically with eyes closed or fixated. The resulting time series exhibit structured temporal correlations among anatomically distinct but functionally connected areas. Spectral analyses have revealed that the majority of functional connectivity arises from low-frequency components, which correspond to the timescale of slow hemodynamic modulations, and are extracted and quantified through methods such as seed-based correlation analysis (SCA), independent component analysis (ICA), or graph-theoretical modeling [7,39,40]. These spontaneous fluctuations are organized into spatially distinct, yet temporally coherent systems known as resting-state networks (RSNs), which are highly reproducible across subjects and sessions, suggesting that they represent stable, physiologically meaningful patterns of functional organization [7].

### 4.2. Default Mode Network, Motor, Auditory, and Language Systems

Resting-state analyses consistently identify a set of robust and reproducible intrinsic connectivity networks that mirror task-activated systems, underscoring their physiological significance [7]. Among these, the DMN is the most extensively characterized. It comprises the ventral medial prefrontal cortex (vMPFC), posterior cingulate cortex (PCC), and bilateral angular gyri, forming a coherent system that shows relative decreases in activity during externally directed, goal-oriented tasks compared with passive or internally focused states [41]. Originally identified through PET meta-analyses of task-induced deactivations, the DMN was later confirmed in fMRI studies as a set of regions exhibiting strong low-frequency functional coupling at rest, particularly between the PCC and vMPFC, which together constitute the core hubs of the network [36,41]. In the model proposed by Buckner et al. [41], the DMN architecture comprises a central Core system (PCC–vMPFC) that integrates two interacting subsystems:

- The Medial Temporal Lobe (MTL) subsystem, associated with episodic memory and mental simulation of past or future events.
- The Dorsal Medial (DM) prefrontal subsystem, involved in social cognition and Theory of Mind processes.

Functionally, the DMN supports self-referential thought, autobiographical memory, and introspection, operating as a foundation for internally oriented cognition. Developmentally, it is one of the last large-scale networks to mature, transitioning from localized connectivity in childhood to the distributed configuration seen in adults, which parallels the emergence of self-awareness and complex social cognition [41]. Clinically, alterations in DMN connectivity and activity have been documented across numerous neurological and psychiatric disorders, including Alzheimer’s disease, traumatic brain injury, schizophrenia, and depression [42,43,44]. In neuro-oncology, DMN disruption and reorganization influenced by a tumor has been linked to impaired cognition and language lateralization [45]. As such, functional assessment of the DMN may serve as a sensitive indicator of large-scale network disruption, supporting surgical planning and prognostic evaluation of post-surgical cognitive recovery.

The SMN comprises the precentral and postcentral gyri, supplementary motor area (SMA), and adjacent parietal cortical regions, forming a bilateral system involved in motor execution and somatosensory processing [35,46]. The SMN was the first intrinsic functional network to be reliably identified using resting-state fMRI. In their seminal study, Biswal et al. demonstrated that spontaneous BOLD signal fluctuations in the left and right motor cortices were highly correlated when participants were at rest, revealing the principle of intrinsic functional connectivity that underlies all resting-state networks [35]. Physiologically, the SMN connectivity patterns closely mirror those elicited during task-based paradigms such as finger tapping or motor imagery, underscoring the stability of functional architecture across rest and activation states [47]. Owing to its spatial reproducibility and signal robustness, the SMN is often used as a benchmark network for validating rs-fMRI preprocessing pipelines and assessing data quality. Clinically, rs-fMRI mapping of the SMN provides a powerful, task-independent approach for motor cortex localization, particularly valuable for patients who cannot perform active movement due to neurological deficits, cognitive impairment, or young age [17]. Functional connectivity within the SMN has demonstrated high test–retest reliability and correspondence with intraoperative electrocortical stimulation results, supporting its integration into presurgical planning workflows [46].

The Auditory Network (AN) is centered around the bilateral superior temporal gyri, including the transverse temporal gyri (Heschl’s gyri), which host the primary auditory cortex, and the superior temporal sulcus, encompassing secondary and associative auditory areas [7,35]. Functionally, the AN supports auditory perception, phonological processing, and multisensory integration, providing a foundation for both linguistic and nonlinguistic auditory cognition [38]. From a clinical perspective, the AN demonstrated altered resting-state auditory connectivity in patients with tinnitus, providing early evidence that rs-fMRI can detect functional dysregulation within sensory cortices in pathological conditions [48]. Consequently, it shows a promising role in presurgical assessment of lesions near the temporal lobe and in the study of functional reorganization following hearing loss or tumor-induced compression.

The language RSN comprises left-lateralized regions including Broca’s area (located in inferior frontal gyrus), Wernicke’s area (posterior superior temporal gyrus), and the middle temporal gyrus [49]. Resting-state functional connectivity between these regions mirrors the structural integrity of the arcuate fasciculus and correlates strongly with task-based measures of language lateralization. Studies in brain tumor patients have shown that rs-fMRI can delineate the language network even when patients exhibit severe aphasia or cannot perform language tasks [19].

Additional RSNs include the visual, dorsal attention, and frontoparietal control networks, each contributing to higher-order cognitive and sensory processing [30,45]. Together, these systems form a cohesive yet dynamically interacting network ensemble that supports the brain’s ongoing readiness to respond to internal and external demands.

### 4.3. Connectivity Analysis

The identification and quantification of resting-state networks rely on various analytical approaches, each designed to capture distinct aspects of functional connectivity. The most commonly employed methods include SCA, ICA, graph theory, regional homogeneity (ReHo), and amplitude of low-frequency fluctuations (ALFF) [39,40]. Each of these approaches captures different dimensions of resting-state brain dynamics—from pairwise temporal correlations (SCA) to spatially independent networks (ICA), topological organization (graph theory), and local synchrony (ReHo) or activity amplitude (ALFF). In clinical practice, hybrid analytic pipelines often combine multiple methods to achieve more comprehensive functional mapping and to improve robustness in patient populations with structural abnormalities or compromised signal quality [40].

#### 4.3.1. Seed-Based Correlation Analysis (SCA)

The SCA, first introduced by Biswal et al., remains the most widely used and conceptually straightforward method for investigating resting-state functional connectivity [39]. It is a hypothesis-driven approach in which a predefined region of interest (ROI), called the seed, is selected based on prior anatomical or functional knowledge. The mean BOLD time series from the seed is extracted and correlated with the seed, typically using Pearson’s correlation coefficient, with the time series of every other voxel in the brain. The resulting correlation map delineates regions that exhibit synchronous low-frequency fluctuations with the seed, thus identifying functionally connected areas. For example, selecting a seed in the left precentral gyrus reliably reveals correlated activity in the right precentral gyrus, reconstructing the sensorimotor network. SCA’s appeal lies in its simplicity, interpretability, and direct clinical applicability, particularly in presurgical mapping of eloquent cortices [39,50]. The method enables targeted assessment of connectivity patterns around specific cortical landmarks, even in patients unable to perform active tasks. However, its results are inherently seed-dependent and the accuracy of the connectivity map relies on the anatomical precision of seed placement and the validity of the a priori hypothesis. Moreover, because SCA measures pairwise temporal correlations, it may overestimate functional integration between distinct but co-activated networks, thereby failing to distinguish between synchronized but functionally independent systems. This limitation parallels that of the GLM in task-based fMRI, where concurrent activations can confound network specificity [51]. Despite limitations, SCA has demonstrated high clinical value in neurosurgical contexts, such as presurgical mapping for tumor resection where Dierker et al. compared rs-fMRI SCA-derived motor maps with conventional task-based fMRI in patients undergoing tumor resection and found high accuracy overlap between SCA maps and anatomical reference standards in the sensorimotor region [50].

#### 4.3.2. Independent Component Analysis (ICA)

The ICA is a data-driven, multivariate analytical technique foundational to rs-fMRI, used to separate the complex BOLD signal into distinct, statistically independent spatial sources [51]. ICA assumes that the observed fMRI signal is a linear mixture of independent sources, such as functional networks, motion, and physiological noise, and derives an unmixing matrix that maximizes statistical independence among components. The result is a set of spatially independent maps and their corresponding time courses, known as independent components (ICs), which can be classified as RSNs or noise components [52,53]. Unlike SCA, ICA does not rely on predefined regions of interest, making it ideal for whole-brain exploration of functional connectivity, which allows ICA to identify all major RSNs simultaneously and to detect novel or atypical connectivity patterns [52]. Importantly, the ICA distinguishes the brain’s enduring intrinsic connectivity from transient, task-modulated connectivity, while SCA cannot. When a task co-activates two intrinsically separate networks, SCA measures the combined, temporary synchronization, revealing the functional connectivity of the brain region for that specific experimental state, while the ICA is able to separate the enduring intrinsic connectivity patterns from the transient, task-modulated changes [51]. A single brain region may be part of several ICA-derived networks, and its functional role is defined by its participation in, and interaction with, these distinct RSNs. A major strength of ICA lies in its ability to separate neural signals from physiological and motion-related artifacts, a feature particularly valuable at ultra-high field strengths, where susceptibility artifacts are amplified [53]. However, a key limitation is the necessity for subsequent component classification, where the extracted ICs must be identified as either signal or noise, which can be complex and sometimes subjective [52]. The number of components to be extracted also must be specified in advance, which influences the resolution of the final networks [51]. The clinical utility of ICA is significant particularly in presurgical mapping for tumors or epilepsy. It can accurately identify and localize functional eloquent cortex even when structural distortion is present, which makes traditional SCA difficult, demonstrating its invaluable role in patient care [53]. A widely adopted framework for performing ICA in neuroimaging is the GIFT (Group ICA of fMRI Toolbox), built on wide range of ICA, which provides wide tools for component visualization, statistical testing, data pre-processing, and artifact identification. It supports advanced functionalities, such as spatial and dynamic ICA, ICASSO component stability analysis, and automatic tools integration, allowing widespread application for classification of cognitive states and neurological disorders [54].

#### 4.3.3. Graph Theory Analysis

Graph theory provides a robust mathematical framework for modeling the brain’s functional architecture as a network, or “connectome,” comprising nodes—typically defined brain regions—and edges that represent functional connections between them. Nodes are delineated using anatomical atlases or functional parcellation techniques such as ICA, while edges quantify statistical relationships, most commonly correlations, between regional time series [55]. The application of graph theory enables efficient computation and comparison of various network topologies within a shared theoretical framework. Technically, these networks are represented as adjacency matrices where each row and column correspond to brain regions and the entries reflect the presence or magnitude of connectivity [39]. Graph theory translates complex patterns of functional connections into a small set of neurobiologically meaningful metrics, which fall broadly into two conceptual categories: functional segregation and functional integration. Functional segregation refers to the tendency of brain regions to form tightly connected local clusters or modules that support specialized processing, captured by metrics such as clustering coefficient, local efficiency, and modularity. Functional integration, on the other hand, describes the brain’s capacity to efficiently transfer information across distant regions, which is assessed using measures like global efficiency and characteristic path length [56]. Unlike traditional SCA that focus on pairwise correlations, graph theory captures whole-brain topological properties, making it particularly useful for identifying critical hub regions using centrality or degree-based measures. These hubs are essential for facilitating information flow and are often susceptible to disruption in neurological disorders [57]. The graph theory has shown significant utility in both research and clinical domains, particularly in tracking disease progression and monitoring responses to interventions such as surgery, medication, or rehabilitation. In neurosurgical contexts, graph theory can identify critical regions and patterns of compensatory reorganization, providing valuable insights into potential outcomes and functional recovery [57,58]. However, differences in brain parcellation, connectivity definitions, and preprocessing steps can all influence the resulting network structure, limiting reproducibility across studies. Binary networks are often used for their simplicity but may oversimplify brain dynamics, while weighted networks preserve connection strength and offer richer insights, though they demand greater methodological consistency [39,58]. Graph theory has evolved into the field of connectomics, where deep learning is increasingly applied to graph representations for disease classification, brain age estimation, and network anomaly detection [56]. Graph Neural Networks (GNNs) are gaining popularity for modeling brain-wide interactions by utilizing message-passing and neighborhood aggregation to capture the high-dimensional, non-linear relationships inherent in multi-modal brain data. These architectures, ranging from Graph Convolutional to Attention Networks, offer superior capabilities in identifying neuroimaging biomarkers and modeling the dynamic, time-varying nature of functional connectivity [59].

#### 4.3.4. Regional Homogeneity (ReHo)

ReHo quantifies the temporal synchronization of the BOLD signal time series among neighboring voxels. It is considered a measure of local functional connectivity or a form of network centrality that characterizes local features of the brain connectome. The method is based on the assumption that if a brain region is functionally active, its constituent voxels should exhibit similar spontaneous BOLD fluctuations over time. Regions with greater homogeneity are thought to be more synchronized and, thus, more functionally coordinated. ReHo is typically calculated using Kendall’s Coefficient of Concordance (KCC), which measures the similarity of the time courses of a given voxel and its nearest neighbors [60]. The resulting ReHo value for a voxel represents the degree of local synchronization, providing a voxel-wise map of local functional activity throughout the brain [61]. ReHo provides a unique window into the local functional interactions within small-scale spatial neighborhoods and, similar to whole-brain ICA, does not require a priori selection of a seed region, making it a relatively data-driven and highly reproducible approach to identify regions with abnormal intrinsic activity [60]. In clinical studies, ReHo alterations have been detected in peritumoral regions, suggesting localized disturbances in spontaneous activity due to infiltration or edema. ReHo maps have been utilized to bridge the gap between neurophysiology and neuropsychology in pediatric brain tumor patients, as localized synchronization deficits have shown significant correlations with multidimensional cognitive indices [33]. Clinical applications of ReHo extend to detecting subthreshold psychiatric conditions, identifying early-stage abnormalities in the anterior cingulate gyrus, and effectively assessing neuroplastic changes following non-pharmacological interventions [61].

#### 4.3.5. Amplitude of Low-Frequency Fluctuations (ALFF) and Fractional ALFF (fALFF)

Amplitude of Low-Frequency Fluctuations (ALFF) and Fractional ALFF (fALFF) are resting-state fMRI metrics that quantify spontaneous neural activity by measuring the power of low-frequency (0.01–0.10 Hz) fluctuations in the BOLD signal within a voxel or brain region. ALFF is computed as the square root of the power spectrum averaged within the low-frequency band, reflecting the intensity of intrinsic neural activity. fALFF normalizes ALFF by dividing the low-frequency power by the total power across a broader frequency range [62]. Both ALFF and fALFF are computed on a voxel-wise basis, generating spatial maps that depict the regional distribution of signal amplitude across the brain. fALFF specifically offers improved sensitivity and specificity by reducing the contribution of non-neuronal noise from large vessels and tissue interfaces. Limitations involve susceptibility to physiological noise and motion sources, and dependence on preprocessing choices such as temporal filtering and motion correction [63]. However, ALFF and fALFF have demonstrated high temporal stability and reproducibility across sessions and populations, supporting their potential utility as biomarkers for a wide range of neurological and psychiatric disorders, such as epilepsy, schizophrenia, and brain tumors [63,64,65]. ALFF analysis can also reveal compensatory hyperactivity in contralesional areas following cortical damage, offering insight into functional reorganization mechanisms [66]. Recent multivariate machine learning pipelines, such as Support Vector Machines (SVM), leverage the high-dimensional interdependencies of ALFF and structural data to identify complex diagnostic “signatures” that univariate methods may overlook. These approaches have achieved classification accuracies exceeding 80% in identifying neurodevelopmental outcomes by training models to recognize global patterns of brain dysfunction rather than isolated regional changes [63].

## 5. Methodological Comparison of Tb-fMRI and Rs-fMRI

Functional MRI can be implemented using either tb-fMRI or rs-fMRI paradigms, each grounded in the BOLD signal but differing substantially in their methodological assumptions, acquisition protocols, analytical strategies, and clinical applications. While tb-fMRI has long served as the clinical standard for functional localization in presurgical planning, rs-fMRI is rapidly gaining ground as a complementary or alternative technique, especially in populations where task performance is unreliable.

### 5.1. Acquisition and Data Processing

The quality, interpretability, and reproducibility of both tb-fMRI and rs-fMRI data critically depend on acquisition parameters and preprocessing strategies. While both techniques rely on gradient-EPI sequences with its single-shot acquisition allowing for the rapid capture of entire brain volume and BOLD signal fluctuations, differences in protocol optimization reflect their distinct analytical goals [4].

Most clinical and research fMRI studies are conducted on 3.0 Tesla (3T) scanners, which provide an optimal balance between spatial resolution, SNR, and susceptibility artifact management [16]. Functional imaging typically uses T_2_*-weighted gradient-echo EPI sequences, sensitive to changes in deoxyhemoglobin concentration. Standard acquisition parameters include a repetition time (TR) of 2000–3000 ms to allow sampling of low-frequency oscillations (<0.1 Hz), an echo time (TE) of 25–35 ms optimized near the T_2_* of gray matter to maximize the BOLD contrast, a flip angle of 80–90°, and a voxel size between 2.5 and 3.5 mm^3^ isotropic corresponding to an effective spatial resolution of approximately 3 mm. Field of view (FOV) commonly spans ~220 × 220 mm^2^ with 30–40 slices covering the entire brain and bandwidth is usually set to 1500–2500 Hz/pixel, minimizing geometric distortions and signal dropout near air-tissue interfaces [67]. In task-based fMRI, each run typically lasts 3–6 min and targets a specific cognitive domain (e.g., motor, language, visual). Stimuli are presented via auditory or visual systems synchronized with image acquisition, and precise timing is crucial for subsequent GLM analysis. Full protocols often span 20–40 min when multiple paradigms are included [17]. In rs-fMRI, a single continuous acquisition of 5–10 min is standard and sufficient to map multiple intrinsic brain networks, including the default mode, sensorimotor, and language systems [13]. TR values around 2500 ms ensure compliance with the Nyquist criterion for detecting low-frequency fluctuations [68]. Participants are instructed to rest with eyes closed or fixated on a cross, and some centers incorporate physiological monitoring for retrospective noise correction [69]. Although higher field strengths (7T) can improve spatial specificity, they also amplify susceptibility artifacts and physiological noise, limiting their routine clinical use. Thus, the majority of clinical fMRI applications, including presurgical mapping, remain standardized at 3T [17].

### 5.2. Preprocessing Pipelines

The transformation of raw BOLD data into meaningful activation or connectivity maps requires a series of preprocessing steps designed to correct artifacts, enhance SNR, and standardize spatial alignment. While task-based and resting-state fMRI share several steps, such as motion correction, slice timing correction, and normalization, their pipelines diverge in scope and emphasis. For task-based fMRI, preprocessing is most commonly performed using SPM, which is a computational framework that introduced and standardized many of the canonical steps in fMRI analysis, including realignment, normalization, and spatial smoothing. It relies heavily on the GLM to generate SPMs, which identify brain regions where BOLD time series correlate with a modeled task response. Its user-friendly interface and modular design make it especially well-suited for clinical and research applications focused on task-evoked activations [70]. Other widely used toolboxes include the FMRIB Software Library (FSL) v5.0 and the Analysis of Functional NeuroImages (AFNI). FSL is a comprehensive open-source suite written primarily in C++, which employs the GLM framework for task analysis via its FEAT (FMRI Expert Analysis Tool) module and is particularly valued for its robust image registration (FLIRT, FNIRT), segmentation, and non-parametric group-level statistics using the randomize tool. In contrast, AFNI is designed for real-time data processing and advanced methodological flexibility. It supports both GLM-based and data-driven approaches, including ICA, and offers powerful scripting capabilities for customized and large-scale batch processing [71].

**Figure 1:**
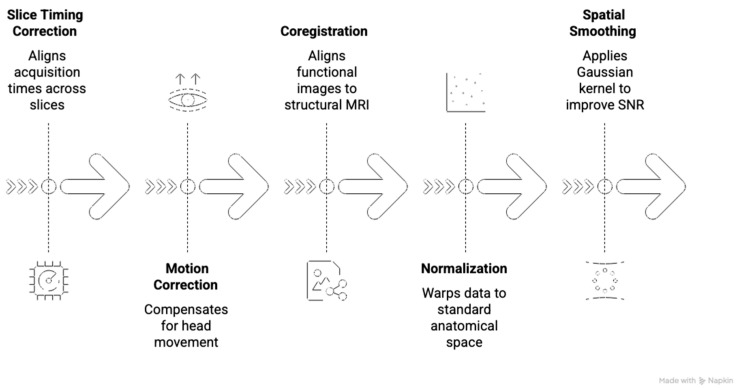
Preprocessing steps for tb-fMRI used in presurgical mapping, including motion correction, anatomical alignment, and spatial smoothing. Accurate execution of these steps ensures reliable localization of eloquent cortex and supports its integration into neuronavigation systems (generated with NapkinAI (https://www.napkin.ai/, accessed 20 December 2025)).

A standard SPM-based preprocessing pipeline consists of five main steps, involving slice timing correction, motion correction, coregistration, normalization, and spatial smoothing, which are also outlined in Figure 1. The process begins with slice timing correction, which aligns acquisition times across slices within a TR to correct for temporal offsets. Motion correction (also known as realignment) is then performed to compensate for involuntary head movement by using rigid-body transformations. These estimated motion parameters are then included as nuisance regressors in the subsequent GLM or connectivity analysis to statistically account for residual motion-related signal variance and if not included, residual motion-related variance may be mistaken for true neural activity, leading to false positives that could jeopardize surgical margins [4]. Next, coregistration is performed to align the lower-resolution functional images to the subject’s high-resolution anatomical T_1_-weighted structural MRI, ensuring functional data is properly mapped onto the individual’s anatomy. Susceptibility artefacts here may cause functional data being projected onto the wrong anatomy, which is a catastrophic error for presurgical planning. The data is then subjected to normalization, a process that warps data into a standard anatomical space, such as the MNI152 template, and is essential for allowing group analysis and for utilization in neuronavigation. The final core step is spatial smoothing, where a Gaussian kernel is applied to the data, serving the dual purpose of improving the SNR and accommodating minor anatomical variability between subjects after normalization [17,72]. Following preprocessing, the data are entered into a GLM where the task design, modeled as a series of stimulus onsets convolved with a canonical hemodynamic response function, is statistically fitted to the observed BOLD signal on a voxel-wise basis [72]. The resulting SPMs reveal localized regions where activation correlates with task performance, forming the basis for functional interpretation and surgical planning.

**Figure 2:**
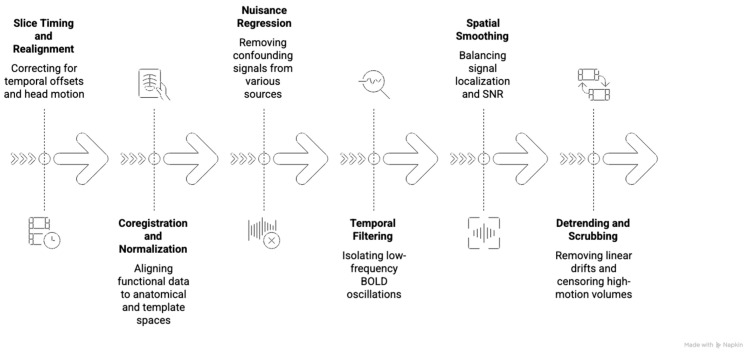
Preprocessing pipeline for rs-fMRI, emphasizing additional denoising steps such as nuisance regression, temporal filtering, detrending, and scrubbing, which are critical for minimizing physiological and motion-related artifacts and enhancing functional connectivity analysis (generated with NapkinAI).

Rs-fMRI requires more extensive preprocessing than tb-fMRI due to its sensitivity to low-frequency BOLD fluctuations and vulnerability to physiological and motion-related artifacts. Several specialized toolboxes have been developed to manage this complexity, many of which are built on MATLAB (https://www.mathworks.com/products/matlab.html, accessed 20 December 2025) and compatible with SPM. DPARSF (Data Processing Assistant for Resting-State fMRI) provides an automated, user-friendly pipeline suitable for batch preprocessing of large datasets and is commonly used to compute standard voxel-wise metrics such as ReHo and ALFF [73]. DPABI (Data Processing & Analysis for Brain Imaging), an extended version of DPARSF, incorporates additional visualization tools, statistical models, and advanced features such as graph-theoretical analysis and more refined noise correction [74]. The CONN Toolbox offers a graphical interface focused on functional connectivity analysis, particularly for seed-based and ROI-to-ROI methods, and is widely favored for its ease of use and robust visualization options in both research and clinical applications [64]. The preprocessing pipeline for rs-fMRI is illustrated on Figure 2. It typically begins with slice timing correction and realignment to adjust for temporal acquisition differences and head motion. Coregistration aligns functional images to the subject’s anatomical scan, followed by normalization into standard space (e.g., MNI), ensuring spatial comparability across subjects. Unlike task-based fMRI, rs-fMRI requires several additional denoising steps [75]. Nuisance regression is used to remove confounding signals from white matter (WM), CSF, and motion parameters, and frequently includes global signal regression, although the latter remains a topic of debate due to its potential to introduce artificial anti-correlations. Temporal band-pass filtering, usually between 0.01 and 0.1 Hz, isolates the low-frequency oscillations that define resting-state networks. Linear trends are removed through detrending, and motion artifacts are mitigated through scrubbing, which censors volumes exceeding a framewise displacement threshold, commonly set at 0.5 mm. Spatial smoothing with a moderate Gaussian kernel is applied to improve SNR while preserving spatial resolution relevant for local connectivity measures [76]. After preprocessing, functional connectivity is computed using seed-based correlation, ICA, or graph-theoretical approaches, depending on the research or clinical objective. Rs-fMRI preprocessing errors, such as inadequate motion scrubbing or skipping nuisance regression for WM or CSF signals, can amplify non-neuronal artifacts, leading to overestimated eloquent cortex volumes that risk unnecessary surgical conservatism or resection into critical networks like sensorimotor areas. In clinical scenarios, global signal regression mistakes may introduce spurious anti-correlations, yielding false-positive connectivity maps that mislocalize tumor margins relative to DCS-validated regions, potentially causing postoperative deficits [77]. Given the susceptibility of rs-fMRI to non-neuronal noise, quality control metrics play a central role in preprocessing and data evaluation. Framewise displacement (FD) quantifies instantaneous head motion between consecutive volumes and helps identify time points that may require censoring. DVARS (temporal derivative of time courses, variance across voxels) detects sudden, widespread signal changes, often related to motion artifacts, while temporal signal-to-noise ratio (tSNR), calculated as the mean signal divided by the temporal standard deviation, serves as a global measure of signal stability across the time series [77,78]. These metrics guide artifact mitigation and are critical for determining the reliability of resting-state connectivity findings.

### 5.3. Motion Correction

Head motion is a major source of noise in fMRI because even submillimeter displacements cause voxel misalignment, spin-history effects, and susceptibility artifacts, all of which distort BOLD signals [78]. In tb-fMRI, motion correction primarily involves rigid-body realignment of each volume to a reference, calculating six motion parameters (3 translations, 3 rotations), which are then included as nuisance regressors in the GLM to remove residual motion-related variance [79]. Motion effects are particularly problematic in paradigms requiring overt responses, such as speech or motor tasks, where task-correlated motion can masquerade as neural activation [75]. Preventive strategies during acquisition, including foam padding, bite-bars, or prospective motion correction using real-time head tracking, can reduce gross movement but do not eliminate subtle, involuntary shifts or interactions with magnetic field gradients. Motion poses an even greater challenge in rs-fMRI, where functional connectivity relies on detecting low-frequency correlations highly susceptible to motion-induced temporal autocorrelations. Movements as small as 0.1 mm can inflate local connectivity and reduce long-range correlations, a bias known as motion-induced distance dependence [80]. To mitigate these effects, preprocessing pipelines incorporate motion quantification and volume censoring. FD measures the instantaneous change in head position between volumes, while DVARS captures sudden global signal changes, and volumes exceeding threshold values (e.g., FD > 0.5 mm) are flagged and removed from analysis [81]. Additionally, data-driven denoising techniques such as ICA-AROMA use ICA to identify and remove signal components correlated with motion, based on spatial and temporal features [81,82]. Despite these methods, residual motion remains a significant confound, particularly in pediatric and clinical populations, necessitating careful quality control and interpretation of connectivity results.

### 5.4. Spatial Smoothing

Spatial smoothing is a standard preprocessing step intended to enhance SNR and accommodate interindividual anatomical variability. It involves convolving the imaging data with a Gaussian kernel to reduce noise and enhance signal detection. The kernel’s width, typically 4–8 mm full width at half maximum (FWHM), determines how much neighboring voxels influence each other’s signal intensities. In task-based fMRI, smoothing improves sensitivity to activation by reducing voxel-wise noise, which helps statistical models like the GLM detect task-related BOLD changes more reliably. It also compensates for anatomical variability across subjects by blurring spatial mismatches, facilitating group-level analyses [76]. However, excessive smoothing can blur activation boundaries, inflating cluster sizes and decreasing spatial accuracy, which is an important trade-off in presurgical mapping where millimeter precision is critical [17]. In rs-fMRI, the role of smoothing is more nuanced. Unlike tb-fMRI, which seeks to identify signal magnitude, resting-state analysis relies on the temporal correlation between voxels; because smoothing mathematically averages the time-series of adjacent voxels, it can artificially force them to appear synchronized. Moderate smoothing (4–6 mm FWHM) may improve reproducibility, but excessive smoothing can inflate short-range correlations and distort the spatial structure of connectivity networks, fundamentally distorting the brain’s intrinsic architectural “fingerprint” [75]. This is especially relevant for voxel-wise connectivity metrics like ReHo or ALFF, where spatial smoothing is often minimized or delayed until after individual-level analyses to preserve fine-grained local signal structure [83]. Therefore, while larger kernels (6–8 mm) are common in tb-fMRI to boost statistical power, rs-fMRI generally favors smaller kernels to avoid masking the subtle, distributed connectivity patterns that define intrinsic brain networks.

### 5.5. Signal Regression

Physiological noise correction is a critical step in fMRI preprocessing, aiming to remove confounding signal fluctuations from non-neuronal sources such as cardiac pulsation, respiration, and slow drifts in CO_2_. These fluctuations often overlap with the low-frequency range of neural BOLD signals, particularly in rs-fMRI, and can obscure true functional connectivity if left uncorrected [4]. The most common approach is regression of signals extracted from WM and CSF regions, which capture non-neuronal fluctuations associated with vascular and respiratory cycles [9]. Motion parameters and their derivatives are also included as nuisance regressors to account for residual motion-related variance. Another widely used method is global signal regression (GSR), which removes the mean BOLD time series across the entire brain. GSR can improve anatomical–functional correspondence and reduce global noise, but it remains controversial due to its potential to introduce artificial anti-correlations and distort network-level metrics, complicating interpretation. The decision to include GSR can alter network connectivity patterns, leading to inconsistent findings and clinical uncertainty when comparing results across studies or patients [84]. Alternative denoising methods such as CompCor (component-based noise correction) extract principal components from WM and CSF time series, offering a more data-driven way to remove physiological noise without global normalization [9]. Advanced pipelines like CONN, DPABI, and fMRIPrep integrate these strategies, allowing flexible denoising approaches that combine physiological noise regressors, motion parameters, and ICA-based artifact removal for optimal data cleaning across populations and paradigms [74,75,85].

### 5.6. Artifact Sensitivity and Post-Processing Controversies

Both task-based and resting-state fMRI are highly susceptible to artifacts from magnetic field inhomogeneities, physiological noise, and preprocessing decisions. Susceptibility artifacts, especially near air-tissue interfaces like the orbitofrontal and temporal regions, cause signal dropout and geometric distortions in both modalities. These effects worsen at higher magnetic field strengths (≥3 Tesla) but can be mitigated using field map corrections, which estimate local magnetic distortions to correct images retrospectively, or multi-echo EPI acquisitions, which combine different echo signals to recover lost data [16]. In tb-fMRI, residual physiological noise is often reduced by temporal filtering, which removes frequencies outside the task-relevant band, or by including baseline conditions in block designs to control for non-task-related variance. Rs-fMRI presents more challenges because physiological fluctuations such as cardiac and respiratory cycles occur within the same low-frequency range (0.01–0.1 Hz) as neural signals, complicating noise separation [17]. RETROICOR (RETROspective Image CORrection) addresses this by modeling physiological noise using simultaneously recorded cardiac and respiratory signals, creating regressors based on the phases of these cycles to remove corresponding variance from each voxel’s time series. Although effective, RETROICOR requires additional physiological recording hardware and signal quality control, limiting its routine clinical use [86]. Methodological choices in post-processing, such as temporal filtering parameters, motion scrubbing, and GSR, vary widely across studies, which limits the comparability of results, meta-analytical synthesis, and generalizability of rs-fMRI findings [75]. To address these issues, standardization initiatives like fMRIPrep and the Brain Imaging Data Structure (BIDS) have been developed, which enable easier sharing and collaboration on preprocessing pipelines [87,88]. However, adopting BIDS can be burdensome, especially in clinical settings, due to the need for specialized tools and meticulous metadata curation. Inaccuracies can disrupt pipelines like fMRIPrep or produce silent errors. Ultimately, while task-based pipelines (e.g., SPM, FSL) have achieved relative consensus over two decades of use, rs-fMRI remains more heterogeneous, with results highly dependent on preprocessing parameters. Even small variations in preprocessing steps, such as motion correction, GSR, and temporal filtering, can substantially alter resting-state connectivity estimates and network topology. A comparison of 14 preprocessing strategies found that the choice of denoising method had a stronger impact on functional connectivity than acquisition parameters themselves [89]. The absence of standardized thresholds for key steps creates major inconsistencies in rs-fMRI findings across sites and pipelines, complicating cross-study comparisons and undermining clinical confidence. Standardization of motion correction thresholds, noise regression schemes, and temporal filters is therefore essential for translating rs-fMRI into robust clinical practice.

### 5.7. Patient Cooperation

The reliance of task-based fMRI on consistent behavioral performance introduces significant vulnerability in clinical populations. Patients with aphasia often cannot engage in language paradigms like word generation or semantic decision tasks, resulting in diminished or absent BOLD responses. Head motion, common in unwell or uncooperative patients, can introduce non-neuronal intensity shifts that overwhelm the small true BOLD fluctuations [69]. Similarly, motor impairments, such as hemiparesis, can render motor paradigms unusable due to an inability to perform physical tasks like finger-tapping or toe movement. In cognitively impaired individuals, including those with dementia, traumatic brain injury, or advanced age, task comprehension and sustained attention are often compromised, leading to variable or incoherent engagement with stimulus blocks [90]. Beyond performance deficits, structural lesions can distort functional topography, leading to pseudo-reorganization of eloquent areas [91]. Compounding these challenges is the fundamental sensitivity of BOLD fMRI to small signal fluctuations [17]. These issues collectively undermine the reliability and reproducibility of tb-fMRI in vulnerable populations. In contrast, resting-state fMRI requires no active task performance, allowing acquisition under minimal patient compliance. Subjects are instructed merely to fixate on a cross or keep their eyes closed. This independence from behavioral execution makes rs-fMRI particularly advantageous in patients with limited cognitive or motor capacity, sedation, or altered consciousness. The ability to map functional networks passively represents a major clinical advantage, particularly in presurgical contexts where reliable localization of the eloquent cortex is essential [69]. Rs-fMRI remains vulnerable to signal alterations arising from tumor-infiltrated regions, including intertwined changes in local and distributed frequency-domain activity, though more advanced approaches are being developed to mitigate these effects [91].

### 5.8. Scan Duration

Scan duration is another important consideration. Task-based fMRI typically requires multiple paradigm runs to map different functional systems (e.g., motor, language, visual), with each run lasting 3–6 min depending on task design [4]. Due to the intrinsically low SNR of the BOLD response, it is essential to perform multiple repeated stimulations during each task-based measurement to ensure robust and statistically reliable signal detection [17]. Comprehensive mapping protocols may therefore extend total scan time to 30–45 min, increasing the risk of motion artifacts and patient fatigue. Resting-state fMRI, in contrast, typically involves a single, uninterrupted acquisition lasting 5–10 min, during which the entire brain’s functional architecture can be assessed [13]. It leverages intrinsic low-frequency fluctuations, which are continuously present and measurable with shorter, uninterrupted scanning [69]. This time efficiency is particularly beneficial in clinical workflows where shorter protocols reduce the burden on patients and improve throughput. While stable connectivity can be well captured in shorter scan time, reliability improves with longer acquisitions of up to 12–13 min [92]. Despite its brevity, rs-fMRI captures stable and reproducible connectivity patterns, provided that data quality and preprocessing are adequately controlled [93].

### 5.9. Robustness to Pathology

Tb-fMRI is particularly susceptible to limitations in patients with tumors, vascular lesions, or other structural abnormalities. Performance deficits can impair task execution, leading to absent or unreliable activation maps, with failure rates as high as 38.5% in challenging cases [94]. Mass effect, edema, and tumor-associated vascular remodeling alter neurovascular coupling in the perilesional cortex, which attenuate or distort the BOLD response, even when the underlying neuronal activity is preserved, which can displace eloquent regions or compromise hemodynamic responsiveness [95,96]. As a result, task-based paradigms may underestimate the extent or location of functional tissue, posing challenges for accurate presurgical planning. Rs-fMRI, by contrast, appears more resilient to such pathological variability. Because it captures intrinsic functional connectivity independent of task performance, it can detect coherent network activity even in structurally distorted or infiltrated tissue [94]. Studies have demonstrated that rs-fMRI successfully delineates sensorimotor and language networks in patients with gliomas, stroke, and epilepsy, achieving around 91% sensitivity and 89% specificity for motor mapping compared to tb-fMRI showing sensitivity of 78–100% and specificity of 46–97% against DCS [19,25,97]. Rs-fMRI also detects tumor-induced network reorganization and compensatory mechanisms more consistently, aiding presurgical planning to accurate resection while preserving function [94]. Beyond localization, rs-fMRI offers insights into functional plasticity by revealing compensatory recruitment of alternative regions or shifts in network topology in response to chronic lesions, which may contribute to a more nuanced understanding of disease burden and recovery potential [19,98]. Nevertheless, rs-fMRI is not immune to confounds. Tumor-induced alterations in vascular reactivity, SNR, and physiological noise can still influence correlation patterns [99]. Moreover, the technique’s sensitivity to head motion and reliance on advanced data processing necessitate rigorous preprocessing and careful clinical interpretation [69,99]. Validation against anatomical landmarks, structural imaging, and intraoperative mapping remains essential to ensure clinical reliability.

### 5.10. Clinical Acceptance

From a clinical standpoint, tb-fMRI remains the gold standard for non-invasive functional localization. Its paradigm-driven nature and extensive validation against intraoperative cortical stimulation have established its diagnostic reliability, particularly in presurgical motor and language mapping [17]. Clinical guidelines from major neuroimaging societies continue to recognize tb-fMRI as an accepted adjunct to neuronavigation in brain tumor and epilepsy surgery [100]. However, clinical adoption of rs-fMRI is increasing rapidly. Its task independence, time efficiency, and ability to reveal multiple networks in a single scan make it highly attractive for clinical translation [94]. Several studies have reported high spatial concordance (80–90%) between rs-fMRI-derived network maps and task-based activation patterns, as well as with DCS [19,26]. Rs-fMRI lacks standardized thresholds for activation strength and validated specificity compared to tb-fMRI, especially in cases of atypical anatomy or compromised vascular reactivity where BOLD signals may be biased [69]. Vascular reactivity compromises, common in cerebrovascular disease, can confound low-frequency BOLD fluctuations, reducing reliability in patients with gliomas or stroke [94,101]. However, clinical adoption is growing, with hundreds of cases showing DCS-validated results, signaling readiness for broader use. Ongoing advancements, like ICA and neural networks, position rs-fMRI as a reliable alternative, with many studies affirming its preoperative utility and lower failure rates (13% vs. 38.5% for tb-fMRI) [94]. Rs-fMRI can provide essential functional insights without additional patient burden or compliance requirements in patients unable to perform tasks reliably, such as those with cognitive impairment, pediatric populations, or individuals with language barriers or motor deficits. Looking ahead, there is a growing need for multicenter validation studies, harmonized workflows, and consensus guidelines to ensure reproducibility, improve interpretability, and support the transition of rs-fMRI from research into routine clinical practice. The emerging consensus suggests a complementary use of both modalities: tb-fMRI for direct, hypothesis-driven localization of specific functions, and rs-fMRI for comprehensive, task-independent mapping of functional connectivity. Together, they provide a multidimensional understanding of the brain’s functional landscape, enhancing the precision and safety of neurosurgical interventions [50,69].

As a narrative review, this study is inherently limited by its non-systematic methodology. Although we aimed for comprehensive coverage, it is possible that some relevant studies were overlooked or unevenly represented, and methodological differences across included works may not be fully captured. Therefore, findings should be interpreted with awareness of these constraints.

## 6. Conclusions

As fMRI continues to evolve as a critical tool in presurgical brain mapping, rs-fMRI rs-fMRI is emerging as a strong complementary or alternative approach, especially valuable in patients unable to perform tasks due to cognitive, motor, or language impairments. This review has highlighted the fundamental principles of the BOLD signal and its hemodynamic underpinnings, contrasted the acquisition and preprocessing pipelines of tb-fMRI and rs-fMRI, and explored their respective strengths, limitations, and clinical applications. While rs-fMRI offers broader accessibility and network-level insights in a time-efficient manner, its clinical translation is challenged by variability in preprocessing decisions, which can significantly influence reliability and comparability across studies. There is growing evidence for the spatial concordance of rs-fMRI with tb-fMRI and direct DCS, and promising results in mapping functional networks even in the presence of tumors or vascular pathology. However, the lack of standardized protocols, validated thresholds, and interpretive frameworks remains a barrier to broader clinical adoption. Progress in this field depends on multicenter validation studies, harmonized acquisition and preprocessing standards, and the development of consensus-based clinical guidelines. The integration of advanced techniques, such as machine learning, connectomics, and preprocessing tools, may enhance the interpretability of rs-fMRI and support its adoption as a routine clinical tool. Combining the strengths of both task-based and resting-state approaches can provide a multidimensional perspective on brain function, improving surgical planning, patient safety, and neurological outcomes.

## Data Availability

No new data were created or analyzed in this study. Data sharing is not applicable to this article.

## Abbreviations

The following abbreviations are used in this manuscript:

ALFF	Amplitude of Low-Frequency Fluctuations
AN	Auditory Network
AFNI	Analysis of Functional NeuroImages
BIDS	Brain Imaging Data Structure
BOLD	Blood Oxygenation Level–Dependent
CBF	Cerebral Blood Flow
CBV	Cerebral Blood Volume
CMRO_2_	Cerebral Metabolic Rate of Oxygen
CO_2_	Carbon Dioxide
CONN	CONN Functional Connectivity Toolbox
CSF	Cerebrospinal Fluid
DCS	Direct Cortical Stimulation
dHb	Deoxygenated Hemoglobin
DM	Dorsal Medial
DMN	Default Mode Network
DPABI	Data Processing & Analysis for Brain Imaging
DPARSF	Data Processing Assistant for Resting-State fMRI
DVARS	Temporal Derivative of Time Courses, Variance Across Voxels
EPI	Echo-Planar Imaging
fALFF	Fractional Amplitude of Low-Frequency Fluctuations
FD	Framewise Displacement
FDR	False Discovery Rate
FEAT	FMRI Expert Analysis Tool
FLIRT	FMRIB’s Linear Image Registration Tool
FMRIB	Functional Magnetic Resonance Imaging of the Brain
FNIRT	FMRIB’s Nonlinear Image Registration Tool
FOV	Field of View
fMRI	Functional Magnetic Resonance Imaging
FSL	FMRIB Software Library
FWE	Family-Wise Error
GLM	General Linear Model
GNN	Graph Neural Networks
GSR	Global Signal Regression
Hb	Hemoglobin
HRF	Hemodynamic Response Function
ICA	Independent Component Analysis
ICA-AROMA	ICA-based Automatic Removal Of Motion Artifacts
ICs	Independent Components
KCC	Kendall’s Coefficient of Concordance
LFPs	Local Field Potentials
MTL	Medial Temporal Lobe
MUA	Multiunit Activity
NO	Nitric Oxide
OEF	Oxygen Extraction Fraction
PCC	Posterior Cingulate Cortex
PET	Positron Emission Tomography
PLS	Partial Least Squares
ReHo	Regional Homogeneity
ROI	Region of Interest
Rs-fMRI	Resting-State Functional Magnetic Resonance Imaging
RSN	Resting-State Network
SCA	Seed-Based Correlation Analysis
SMN	Sensorimotor Network
SMA	Supplementary Motor Area
SNR	Signal-to-Noise Ratio
SPM	Statistical Parametric Mapping
SVM	Support Vector Machine
sLFFs	Spontaneous Low-Frequency Fluctuations
Tb-fMRI	Task-Based Functional Magnetic Resonance Imaging
TE	Echo Time
TR	Repetition Time
vACC	Ventral Anterior Cingulate Cortex
vMPFC	Ventral Medial Prefrontal Cortex
WM	White Matter

## References

[1] Logothetis N.K. (2008). What We Can Do and What We Cannot Do with fMRI. Nature.

[2] Bandettini P.A. (2012). Twenty Years of Functional MRI: The Science and the Stories. Neuroimage.

[3] Buxton R.B. (2013). The Physics of Functional Magnetic Resonance Imaging (fMRI). Rep. Prog. Phys..

[4] Huettel S.A., Song A.W., McCarthy G. (2004). Functional Magnetic Resonance Imaging.

[5] Heeger D.J., Ress D. (2002). What Does fMRI Tell Us about Neuronal Activity?. Nat. Rev. Neurosci..

[6] Friston K.J., Jezzard P., Turner R. (1994). Analysis of Functional MRI Time-series. Hum. Brain Mapp..

[7] Fox M.D., Raichle M.E. (2007). Spontaneous Fluctuations in Brain Activity Observed with Functional Magnetic Resonance Imaging. Nat. Rev. Neurosci..

[8] Leuthardt E.C., Guzman G., Bandt S.K., Hacker C., Vellimana A.K., Limbrick D., Milchenko M., Lamontagne P., Speidel B., Roland J. (2018). Integration of Resting State Functional MRI into Clinical Practice—A Large Single Institution Experience. PLoS ONE.

[9] Muschelli J., Nebel M.B., Caffo B.S., Barber A.D., Pekar J.J., Mostofsky S.H. (2014). Reduction of Motion-Related Artifacts in Resting State fMRI Using aCompCor. Neuroimage.

[10] Ogawa S., Lee T.M., Kay A.R., Tank D.W. (1990). Brain Magnetic Resonance Imaging with Contrast Dependent on Blood Oxygenation. Proc. Natl. Acad. Sci. USA.

[11] Uludağ K. (2023). Physiological Modeling of the BOLD Signal and Implications for Effective Connectivity: A Primer. Neuroimage.

[12] Chen G., Taylor P.A., Reynolds R.C., Leibenluft E., Pine D.S., Brotman M.A., Pagliaccio D., Haller S.P. (2023). BOLD Response Is More than Just Magnitude: Improving Detection Sensitivity through Capturing Hemodynamic Profiles. NeuroImage.

[13] Damoiseaux J.S., Rombouts S.A.R.B., Barkhof F., Scheltens P., Stam C.J., Smith S.M., Beckmann C.F. (2006). Consistent Resting-State Networks across Healthy Subjects. Proc. Natl. Acad. Sci. USA.

[14] Logothetis N.K., Wandell B.A. (2004). Interpreting the BOLD Signal. Annu. Rev. Physiol..

[15] Lippert M.T., Steudel T., Ohl F., Logothetis N.K., Kayser C. (2010). Coupling of Neural Activity and fMRI-BOLD in the Motion Area MT. Magn. Reson. Imaging.

[16] Jezzard P., Clare S. (1999). Sources of Distortion in Functional MRI Data. Hum. Brain Mapp..

[17] Stippich C. (2015). Clinical Functional MRI: Presurgical Functional Neuroimaging.

[18] Blockley N.P., Griffeth V.E.M., Simon A.B., Buxton R.B. (2013). A Review of Calibrated Blood Oxygenation Level-Dependent (BOLD) Methods for the Measurement of Task-Induced Changes in Brain Oxygen Metabolism. NMR Biomed..

[19] Rolinski R., You X., Gonzalez-Castillo J., Norato G., Reynolds R.C., Inati S.K., Theodore W.H. (2020). Language Lateralization from Task-based and Resting State Functional MRI in Patients with Epilepsy. Hum. Brain Mapp..

[20] Visscher K.M., Miezin F.M., Kelly J.E., Buckner R.L., Donaldson D.I., McAvoy M.P., Bhalodia V.M., Petersen S.E. (2003). Mixed Blocked/Event-Related Designs Separate Transient and Sustained Activity in fMRI. Neuroimage.

[21] Worsley K.J., Friston K.J. (1995). Analysis of fMRI Time-Series Revisited--Again. Neuroimage.

[22] Nichols T., Hayasaka S. (2003). Controlling the Familywise Error Rate in Functional Neuroimaging: A Comparative Review. Stat. Methods Med. Res..

[23] Petrella J.R., Shah L.M., Harris K.M., Friedman A.H., George T.M., Sampson J.H., Pekala J.S., Voyvodic J.T. (2006). Preoperative Functional MR Imaging Localization of Language and Motor Areas: Effect on Therapeutic Decision Making in Patients with Potentially Resectable Brain Tumors. Radiology.

[24] Huang S., De Brigard F., Cabeza R., Davis S.W. (2024). Connectivity Analyses for Task-Based fMRI. Phys. Life Rev..

[25] Morrison M.A., Tam F., Garavaglia M.M., Hare G.M.T., Cusimano M.D., Schweizer T.A., Das S., Graham S.J. (2016). Sources of Variation Influencing Concordance between Functional MRI and Direct Cortical Stimulation in Brain Tumor Surgery. Front. Neurosci..

[26] Lemée J.-M., Berro D.H., Bernard F., Chinier E., Leiber L.-M., Menei P., Ter Minassian A. (2019). Resting-State Functional Magnetic Resonance Imaging versus Task-Based Activity for Language Mapping and Correlation with Perioperative Cortical Mapping. Brain Behav..

[27] Rutten G.-J., Ramsey N.F. (2010). The Role of Functional Magnetic Resonance Imaging in Brain Surgery. Neurosurg. Focus.

[28] Drobyshevsky A., Baumann S.B., Schneider W. (2006). A Rapid fMRI Task Battery for Mapping of Visual, Motor, Cognitive, and Emotional Function. Neuroimage.

[29] Lee K.S., Hagan C.N., Hughes M., Cotter G., McAdam Freud E., Kircanski K., Leibenluft E., Brotman M.A., Tseng W.-L. (2023). Systematic Review and Meta-Analysis: Task-Based fMRI Studies in Youths With Irritability. J. Am. Acad. Child. Adolesc. Psychiatry.

[30] Hacker C.D., Roland J.L., Kim A.H., Shimony J.S., Leuthardt E.C. (2019). Resting-State Network Mapping in Neurosurgical Practice: A Review. Neurosurg. Focus.

[31] Soares J.F., Abreu R., Lima A.C., Sousa L., Batista S., Castelo-Branco M., Duarte J.V. (2022). Task-Based Functional MRI Challenges in Clinical Neuroscience: Choice of the Best Head Motion Correction Approach in Multiple Sclerosis. Front. Neurosci..

[32] Al-Arfaj H.K., Al-Sharydah A.M., AlSuhaibani S.S., Alaqeel S., Yousry T. (2023). Task-Based and Resting-State Functional MRI in Observing Eloquent Cerebral Areas Personalized for Epilepsy and Surgical Oncology Patients: A Review of the Current Evidence. J. Pers. Med..

[33] Zhang Z., Wang J., He L., Huang Z., Sun L., Zhang Y., Zhang X. (2024). Assessing Regional Homogeneity and Cognitive Function Alterations in Pediatric Brain Tumor Patients: A Resting-State Functional Magnetic Resonance Imaging Study. Quant. Imaging Med. Surg..

[34] Lakhani D.A., Sabsevitz D.S., Chaichana K.L., Quiñones-Hinojosa A., Middlebrooks E.H. (2023). Current State of Functional MRI in the Presurgical Planning of Brain Tumors. Radiol. Imaging Cancer.

[35] Biswal B., Yetkin F.Z., Haughton V.M., Hyde J.S. (1995). Functional Connectivity in the Motor Cortex of Resting Human Brain Using Echo-Planar MRI. Magn. Reson. Med..

[36] Greicius M.D., Krasnow B., Reiss A.L., Menon V. (2003). Functional Connectivity in the Resting Brain: A Network Analysis of the Default Mode Hypothesis. Proc. Natl. Acad. Sci. USA.

[37] Raichle M.E. (2015). The Brain’s Default Mode Network. Annu. Rev. Neurosci..

[38] Leopold D.A., Maier A. (2012). Ongoing Physiological Processes in the Cerebral Cortex. Neuroimage.

[39] Smitha K.A., Akhil Raja K., Arun K.M., Rajesh P.G., Thomas B., Kapilamoorthy T.R., Kesavadas C. (2017). Resting State fMRI: A Review on Methods in Resting State Connectivity Analysis and Resting State Networks. Neuroradiol. J..

[40] Lee M.H., Smyser C.D., Shimony J.S. (2013). Resting-State fMRI: A Review of Methods and Clinical Applications. AJNR Am. J. Neuroradiol..

[41] Buckner R.L., Andrews-Hanna J.R., Schacter D.L. (2008). The Brain’s Default Network: Anatomy, Function, and Relevance to Disease. Ann. N. Y. Acad. Sci..

[42] Zhang D., Snyder A.Z., Fox M.D., Sansbury M.W., Shimony J.S., Raichle M.E. (2008). Intrinsic Functional Relations Between Human Cerebral Cortex and Thalamus. J. Neurophysiol..

[43] Tan W., Ouyang X., Huang D., Wu Z., Liu Z., He Z., Long Y. (2023). Disrupted Intrinsic Functional Brain Network in Patients with Late-Life Depression: Evidence from a Multi-Site Dataset. J. Affect. Disord..

[44] Cao C., Liu W., Hou C., Chen Y., Liao F., Long H., Chen D., Chen X., Li F., Huang J. (2025). Disrupted Default Mode Network Connectivity and Its Role in Negative Symptoms of Schizophrenia. Psychiatry Res..

[45] Saviola F., Zigiotto L., Novello L., Zacà D., Annicchiarico L., Corsini F., Rozzanigo U., Papagno C., Jovicich J., Sarubbo S. (2022). The Role of the Default Mode Network in Longitudinal Functional Brain Reorganization of Brain Gliomas. Brain Struct. Funct..

[46] Xiong Z., Tian C., Zeng X., Huang J., Wang R. (2020). The Relationship of Functional Connectivity of the Sensorimotor and Visual Cortical Networks Between Resting and Task States. Front. Neurosci..

[47] Witt S.T., Laird A.R., Meyerand M.E. (2008). Functional Neuroimaging Correlates of Finger-Tapping Task Variations: An ALE Meta-Analysis. Neuroimage.

[48] Huang J., Lin J., You N., Li X., Xiong Y., Wang X., Lu H., Li C., Li R., Hu J. (2024). Tumor Characteristics, Brain Functional Activity, and Connectivity of Tinnitus in Patients with Vestibular Schwannoma: A Pilot Study. Quant. Imaging Med. Surg..

[49] Ramage A.E., Aytur S., Ballard K.J. (2020). Resting-State Functional Magnetic Resonance Imaging Connectivity Between Semantic and Phonological Regions of Interest May Inform Language Targets in Aphasia. J. Speech Lang. Hear. Res..

[50] Dierker D., Roland J.L., Kamran M., Rutlin J., Hacker C.D., Marcus D.S., Milchenko M., Miller-Thomas M.M., Benzinger T.L., Snyder A.Z. (2017). Resting-State Functional Magnetic Resonance Imaging in Presurgical Functional Mapping: Sensorimotor Localization. Neuroimaging Clin. N. Am..

[51] Joel S.E., Caffo B.S., van Zijl P.C.M., Pekar J.J. (2011). On the Relationship between Seed-Based and ICA-Based Measures of Functional Connectivity. Magn. Reson. Med..

[52] Beckmann C.F., DeLuca M., Devlin J.T., Smith S.M. (2005). Investigations into Resting-State Connectivity Using Independent Component Analysis. Philos. Trans. R. Soc. Lond. B Biol. Sci..

[53] Robinson S.D., Schöpf V., Cardoso P., Geissler A., Fischmeister F.P.S., Wurnig M., Trattnig S., Beisteiner R. (2013). Applying Independent Component Analysis to Clinical fMRI at 7 T. Front. Hum. Neurosci..

[54] Iraji A., Faghiri A., Lewis N., Fu Z., Rachakonda S., Calhoun V.D. (2021). Tools of the Trade: Estimating Time-Varying Connectivity Patterns from fMRI Data. Soc. Cogn. Affect. Neurosci..

[55] Vecchio F., Miraglia F., Maria Rossini P. (2017). Connectome: Graph Theory Application in Functional Brain Network Architecture. Clin. Neurophysiol. Pract..

[56] Hassett J.D., Craig B.T., Hilderley A., Kinney-Lang E., Yeates K.O., MacMaster F.P., Miller J., Noel M., Brooks B.L., Barlow K. (2024). Development of the Whole-Brain Functional Connectome Explored via Graph Theory Analysis. Aperture Neuro.

[57] Tanglay O., Dadario N.B., Chong E.H.N., Tang S.J., Young I.M., Sughrue M.E. (2023). Graph Theory Measures and Their Application to Neurosurgical Eloquence. Cancers.

[58] Farahani F.V., Karwowski W., Lighthall N.R. (2019). Application of Graph Theory for Identifying Connectivity Patterns in Human Brain Networks: A Systematic Review. Front. Neurosci..

[59] Mohammadi H., Karwowski W. (2024). Graph Neural Networks in Brain Connectivity Studies: Methods, Challenges, and Future Directions. Brain Sci..

[60] Jiang L., Zuo X.-N. (2016). Regional Homogeneity: A Multimodal, Multiscale Neuroimaging Marker of the Human Connectome. Neuroscientist.

[61] Shen W., Wang X., Li Q., Ding Q., Zhang H., Qian Z., Sun Z., Chen X., Zhang J., Zhao M. (2024). Research on Adults with Subthreshold Depression after Aerobic Exercise: A Resting-State fMRI Study Based on Regional Homogeneity (ReHo). Front. Neurosci..

[62] Jia X.-Z., Sun J.-W., Ji G.-J., Liao W., Lv Y.-T., Wang J., Wang Z., Zhang H., Liu D.-Q., Zang Y.-F. (2020). Percent Amplitude of Fluctuation: A Simple Measure for Resting-State fMRI Signal at Single Voxel Level. PLoS ONE.

[63] Shang J., Fisher P., Bäuml J.G., Daamen M., Baumann N., Zimmer C., Bartmann P., Boecker H., Wolke D., Sorg C. (2019). A Machine Learning Investigation of Volumetric and Functional MRI Abnormalities in Adults Born Preterm. Hum. Brain Mapp..

[64] Golestani A.M., Kwinta J.B., Khatamian Y.B., Chen J.J. (2017). The Effect of Low-Frequency Physiological Correction on the Reproducibility and Specificity of Resting-State fMRI Metrics: Functional Connectivity, ALFF, and ReHo. Front. Neurosci..

[65] Turner J.A., Damaraju E., Van Erp T.G.M., Mathalon D.H., Ford J.M., Voyvodic J., Mueller B.A., Belger A., Bustillo J., McEwen S.C. (2013). A Multi-Site Resting State fMRI Study on the Amplitude of Low Frequency Fluctuations in Schizophrenia. Front. Neurosci..

[66] Shi S., Meng J., Wu X., Wang J., Wang H., Li P., Qie S. (2025). The Relationship between Fractional Amplitude of Low-Frequency Fluctuations (fALFF) and the Severity of Neglect in Patients with Unilateral Spatial Neglect (USN) after Stroke: A Functional near-Infrared Spectroscopy Study. IBRO Neurosci. Rep..

[67] Ogawa S., Menon R.S., Tank D.W., Kim S.G., Merkle H., Ellermann J.M., Ugurbil K. (1993). Functional Brain Mapping by Blood Oxygenation Level-Dependent Contrast Magnetic Resonance Imaging. A Comparison of Signal Characteristics with a Biophysical Model. Biophys. J..

[68] Cordes D., Haughton V.M., Arfanakis K., Carew J.D., Turski P.A., Moritz C.H., Quigley M.A., Meyerand M.E. (2001). Frequencies Contributing to Functional Connectivity in the Cerebral Cortex in “Resting-State” Data. AJNR Am. J. Neuroradiol..

[69] Barkhof F., Haller S., Rombouts S.A.R.B. (2014). Resting-State Functional MR Imaging: A New Window to the Brain. Radiology.

[70] Friston K.J. (2007). Statistical Parametric Mapping: The Analysis of Funtional Brain Images.

[71] Pauli R., Bowring A., Reynolds R., Chen G., Nichols T.E., Maumet C. (2016). Exploring fMRI Results Space: 31 Variants of an fMRI Analysis in AFNI, FSL, and SPM. Front. Neuroinform..

[72] Nichols T.E., Holmes A.P. (2002). Nonparametric Permutation Tests for Functional Neuroimaging: A Primer with Examples. Hum. Brain Mapp..

[73] Chao-Gan Y., Yu-Feng Z. (2010). DPARSF: A MATLAB Toolbox for “Pipeline” Data Analysis of Resting-State fMRI. Front. Syst. Neurosci..

[74] Yan C.-G., Wang X.-D., Zuo X.-N., Zang Y.-F. (2016). DPABI: Data Processing & Analysis for (Resting-State) Brain Imaging. Neuroinformatics.

[75] Churchill N.W., Oder A., Abdi H., Tam F., Lee W., Thomas C., Ween J.E., Graham S.J., Strother S.C. (2012). Optimizing Preprocessing and Analysis Pipelines for Single-Subject fMRI. I. Standard Temporal Motion and Physiological Noise Correction Methods. Hum. Brain Mapp..

[76] Mikl M., Marecek R., Hlustík P., Pavlicová M., Drastich A., Chlebus P., Brázdil M., Krupa P. (2008). Effects of Spatial Smoothing on fMRI Group Inferences. Magn. Reson. Imaging.

[77] Kumar V.A., Lee J., Liu H.-L., Allen J.W., Filippi C.G., Holodny A.I., Hsu K., Jain R., McAndrews M.P., Peck K.K. (2024). Recommended Resting-State fMRI Acquisition and Preprocessing Steps for Preoperative Mapping of Language and Motor and Visual Areas in Adult and Pediatric Patients with Brain Tumors and Epilepsy. AJNR Am. J. Neuroradiol..

[78] Power J.D., Barnes K.A., Snyder A.Z., Schlaggar B.L., Petersen S.E. (2012). Spurious but Systematic Correlations in Functional Connectivity MRI Networks Arise from Subject Motion. Neuroimage.

[79] Friston K.J., Ashburner J., Frith C.D., Poline J.-B., Heather J.D., Frackowiak R.S.J. (1995). Spatial Registration and Normalization of Images. Hum. Brain Mapp..

[80] Satterthwaite T.D., Wolf D.H., Loughead J., Ruparel K., Elliott M.A., Hakonarson H., Gur R.C., Gur R.E. (2012). Impact of In-Scanner Head Motion on Multiple Measures of Functional Connectivity: Relevance for Studies of Neurodevelopment in Youth. Neuroimage.

[81] Power J.D., Mitra A., Laumann T.O., Snyder A.Z., Schlaggar B.L., Petersen S.E. (2014). Methods to Detect, Characterize, and Remove Motion Artifact in Resting State fMRI. Neuroimage.

[82] Pruim R.H.R., Mennes M., van Rooij D., Llera A., Buitelaar J.K., Beckmann C.F. (2015). ICA-AROMA: A Robust ICA-Based Strategy for Removing Motion Artifacts from fMRI Data. Neuroimage.

[83] Zang Y., Jiang T., Lu Y., He Y., Tian L. (2004). Regional Homogeneity Approach to fMRI Data Analysis. Neuroimage.

[84] Fox M.D., Zhang D., Snyder A.Z., Raichle M.E. (2009). The Global Signal and Observed Anticorrelated Resting State Brain Networks. J. Neurophysiol..

[85] Whitfield-Gabrieli S., Nieto-Castanon A. (2012). Conn: A Functional Connectivity Toolbox for Correlated and Anticorrelated Brain Networks. Brain Connect..

[86] Glover G.H., Li T.Q., Ress D. (2000). Image-Based Method for Retrospective Correction of Physiological Motion Effects in fMRI: RETROICOR. Magn. Reson. Med..

[87] Esteban O., Markiewicz C.J., Blair R.W., Moodie C.A., Isik A.I., Erramuzpe A., Kent J.D., Goncalves M., DuPre E., Snyder M. (2019). fMRIPrep: A Robust Preprocessing Pipeline for Functional MRI. Nat. Methods.

[88] Gorgolewski K.J., Auer T., Calhoun V.D., Craddock R.C., Das S., Duff E.P., Flandin G., Ghosh S.S., Glatard T., Halchenko Y.O. (2016). The Brain Imaging Data Structure, a Format for Organizing and Describing Outputs of Neuroimaging Experiments. Sci. Data.

[89] Ciric R., Wolf D.H., Power J.D., Roalf D.R., Baum G.L., Ruparel K., Shinohara R.T., Elliott M.A., Eickhoff S.B., Davatzikos C. (2017). Benchmarking of Participant-Level Confound Regression Strategies for the Control of Motion Artifact in Studies of Functional Connectivity. Neuroimage.

[90] Kim H., Kim H.-K., Kim N., Nam C.S. (2021). Dual Task Effects on Speed and Accuracy During Cognitive and Upper Limb Motor Tasks in Adults With Stroke Hemiparesis. Front. Hum. Neurosci..

[91] Falcó-Roget J., Cacciola A., Sambataro F., Crimi A. (2024). Functional and Structural Reorganization in Brain Tumors: A Machine Learning Approach Using Desynchronized Functional Oscillations. Commun. Biol..

[92] Birn R.M., Molloy E.K., Patriat R., Parker T., Meier T.B., Kirk G.R., Nair V.A., Meyerand M.E., Prabhakaran V. (2013). The Effect of Scan Length on the Reliability of Resting-State fMRI Connectivity Estimates. Neuroimage.

[93] Shehzad Z., Kelly A.M.C., Reiss P.T., Gee D.G., Gotimer K., Uddin L.Q., Lee S.H., Margulies D.S., Roy A.K., Biswal B.B. (2009). The Resting Brain: Unconstrained yet Reliable. Cereb. Cortex.

[94] Mhanna H.Y.A., Omar A.F., Radzi Y.M., Oglat A.A., Akhdar H.F., Ewaidat H.A., Almahmoud A., Yaseen A.-B.B., Badarneh L.A., Alhamad O. (2025). Systematic Review of Functional Magnetic Resonance Imaging (fMRI) Applications in the Preoperative Planning and Treatment Assessment of Brain Tumors. Heliyon.

[95] Pillai J.J., Zacà D. (2012). Comparison of BOLD Cerebrovascular Reactivity Mapping and DSC MR Perfusion Imaging for Prediction of Neurovascular Uncoupling Potential in Brain Tumors. Technol. Cancer Res. Treat..

[96] Hou B.L., Bradbury M., Peck K.K., Petrovich N.M., Gutin P.H., Holodny A.I. (2006). Effect of Brain Tumor Neovasculature Defined by rCBV on BOLD fMRI Activation Volume in the Primary Motor Cortex. Neuroimage.

[97] Rosazza C., Aquino D., D’Incerti L., Cordella R., Andronache A., Zacà D., Bruzzone M.G., Tringali G., Minati L. (2014). Preoperative Mapping of the Sensorimotor Cortex: Comparative Assessment of Task-Based and Resting-State FMRI. PLoS ONE.

[98] Fox M.D., Snyder A.Z., Vincent J.L., Raichle M.E. (2007). Intrinsic Fluctuations within Cortical Systems Account for Intertrial Variability in Human Behavior. Neuron.

[99] Liu T.T. (2013). Neurovascular Factors in Resting-State Functional MRI. Neuroimage.

[100] American College of Radiology (2021). Practice Parameter for the Performance of Functional Magnetic Resonance Imaging (fMRI) of the Brain.

[101] Moia S., Chen G., Uruñuela E., Stickland R.C., Termenon M., Caballero-Gaudes C., Bright M.G. (2024). Individual Variability in the Relationship Between Physiological and Resting-State fMRI Metrics. bioRxiv.

